# Rate and factors for scabies recurrence in children in Saudi Arabia: a retrospective study

**DOI:** 10.1186/s12887-019-1565-9

**Published:** 2019-06-08

**Authors:** Anwar E. Ahmed, Hoda Jradi, Doaa A. AlBuraikan, Bashayr I. ALMuqbil, Monirah A. Albaijan, Ali M. Al-Shehri, Hamdan AL-Jahdali

**Affiliations:** 1 King Saud bin Abdulaziz University for Health Sciences, Riyadh, Saudi Arabia; 2 King Abdullah International Medical Research Center, Riyadh, Saudi Arabia; 3 Ministry of National Guard-Health Affairs, Riyadh, Saudi Arabia

**Keywords:** Scabies, Climate factors, Re-infestation, Children, Saudi Arabia

## Abstract

**Background:**

Despite the fact that several scabies outbreaks emerged in schools in Saudi Arabia in 2018, no study has investigated the risk of scabies recurrence among children in Saudi Arabia. This study aimed to estimate the rate of scabies recurrence and identify factors that were associated with an increased risk of recurrence among children.

**Methods:**

This is a multi-center retrospective study of children (age < 14 years) who were diagnosed between May 20, 2015 and September 12, 2018 with one or multiple recurrent scabies at the Ministry of National Guard Health Affairs (MNGHA) hospitals and clinics in Saudi Arabia. Data were obtained from an electronic health system, BestCare database.

**Results:**

A sample of 264 children analyzed (mean age of 6.7 years) resulted in a cumulative number of 316 scabies diagnoses in which 86 (27.2%) experienced scabies recurrence (at least once). Independent factors associated with a high risk of scabies recurrence: older children (adjusted hazard ratio [aHR], 1.036; 95% CI, 1.002–1.072; *P* = 0.039), female gender (aHR, 1.734; 95% CI, 1.329–2.262; *P* = 0.001), Western region of Saudi Arabia (aHR, 1.548; 95% CI, 1.115–2.151; *P* = 0.009), and 2nd tertile season [May to August] (aHR, 2.368; 95% CI, 1.706–3.288; P = 0.001).

**Conclusions:**

The study demonstrated that the recurrence rate of scabies among children is high. Older children, the female gender, the Western region of Saudi Arabia, and the seasonality were independently associated with an increased risk of scabies recurrence. High temperature and low humidity should be explored as leading factors for scabies infestations in Saudi Arabia. Findings derived from this study may be useful for clinicians and governments in optimizing clinical management of scabies cases and contacts.

## Background

Scabies, a highly contagious skin disease [1], recently revealed a disturbing potential for rapid transmissibility among school children in Saudi Arabia. Scabies is not a new disease to Saudi Arabia, but it has been reported in communities with limited epidemic infections. Scabies in Saudi Arabia is uncommonly reported in the literature, e.g., in 2000, scabies was observed among 18 workers living in a crowded residential area [2].

In the first half of 2018, according to the Saudi Ministry of Health, scabies outbreaks with more than 1700 new cases were observed in schools in Mecca, in the Western region of Saudi Arabia. The Saudi Ministry of Health continues to report new cases of scabies outside of the Mecca area. These outbreaks remain undocumented in the literature.

Scabies has been linked to morbidity [3, 4] and may result in tremendous health system [5], public health [6], and economic [5] burdens. Data on the recurrence rate of scabies were limited, as it has not been reported in most countries. General practitioners in France observed a recurrence rate of 25% [7]. A total of 153 patients and hospital staff members in Japan reported a recurrence rate of 32.7% [8]. Inadequate treatment for contacts may lead to re-infestation [9], which results in an increasing recurrence rate of scabies.

Scabies remains a major burden to the health system in Saudi Arabia due to its rapid spread in poor living conditions and overcrowded settings [2]. Studies on evaluation of recurrence of scabies and its associated factors are needed in Saudi Arabia and other countries to establish an effective clinical management of the cases and contacts.

The study investigated a number of hypotheses that geographical and seasonal variations and demographic profile may contribute to high risk of scabies recurrence among children in Saudi Arabia. This study used the Ministry of National Guard Health Affairs (MNGHA) database across Saudi Arabia to estimate the recurrence rate of scabies among children who experienced scabies between May 20, 2015 and September 12, 2018 and identify factors that were associated with a high risk of scabies recurrence.

## Methods

We conducted a multi-center retrospective study of children who were diagnosed between May 20, 2015 and September 12, 2018 with one or multiple recurrent scabies at the MNGHA hospitals and clinics, Saudi Arabia. The study was approved by the Institutional Review Board (IRB) of the MNGHA, Research Protocol # RC18/220/R. Due to the nature of the study design, the study was exempted from informed consent and permission was obtained from the Ministry of National Guard - Health Affairs to access patient data.

Scabies diagnosis was based on clinical examinations with the presence of the following: “scabies burrows,” “typical lesions affecting male genitalia,” or “typical lesions in a typical distribution and two history features” [10, 11]. Microscopy was used to confirm some of the cases. An inclusion criterion was subjects with an age of less than 14 years who were diagnosed with one or multiple episodes of scabies during the study period. We excluded cases reported in outbreaks occurred in the first half year of 2018 in schools in Mecca, Western Saudi Arabia to prevent potential bias.

Data were extracted from unified BESTCare database, a large multi-center electronic health information system implemented in MNGHA in 2015 [12]. BESTCare provides patient-centered care through a single electronic health system accessible to health care providers for documentation and updating records and fully accessible to patients to review their medical records electronically [13]. We retrieved data on children’s age, gender, region where a case was diagnosed, and diagnosis weekdays (Yes/No). In order to describe seasonal patterns we classified time of diagnosis into three tertiles: 1st (January to April), 2nd (May to August), and 3rd (September to December). We gathered data on the clinics where children received their diagnoses: emergency room, family medicine, dermatology, pediatrics, and satellite clinic. The study outcome was timed to the first diagnosis of scabies and to each subsequent diagnosis (if any) of scabies. However, one or multiple scabies diagnoses were observed for each child. A total of 316 scabies diagnoses were identified during the study period.

### Statistical analysis

Data analysis was performed using SAS package Version 9.4 (SAS Institute, Inc., Cary, NC). Sample characteristics (Table 1) were summarized as frequency (n) and percent (%). Children’s age was summarized using mean and standard deviation (±SD) and range. Due to the sequential episodes of scabies observed in our data, recurrent scabies was analyzed using Cox proportional hazards model (CPH) for multiple events. This approach assumes that scabies recurrences within a child are independent [14]. CPH bivariate analysis was used to identify individual factors that were associated with high risk of scabies recurrence (Table 2). CPH multivariate analysis was used to identify factors that were independently associated with a high risk of scabies recurrence (Table 2). A *p*-value (P) ≤ 0.05 (2-tailed) was considered as statistically significant in all analyses. Both unadjusted and adjusted hazard ratio [HR, aHR] with a 95% confidence interval [CI] were used to assess the strength of association.

**Table 1:** Children’s characteristics (*N* = 264)

Characteristics	*Levels*	*n*	%
Gender	*Male*	145	54.9
	*Female*	119	45.1
Region	*Western*	84	31.8
	*Central*	124	47.0
	*Other*	56	21.2
Tertile	*1st*	97	36.7
	*2nd*	109	41.3
	*3rd*	58	22.0
Weekdays	*Yes*	241	91.3
	*No*	23	8.7
Family Medicine	*Yes*	90	34.1
	*No*	174	65.9
Dermatology	*Yes*	18	6.8
	*No*	246	93.2
Emergency room	*Yes*	78	29.5
	*No*	186	70.5
Pediatric	*Yes*	46	17.4
	*No*	218	82.6
Satellite	*Yes*	12	4.5
	*No*	252	95.5

**Table 2:** Individual factors associated with high risk of scabies recurrence

Factor	*Levels*	B	SE	Chi-Square	P	HR	95% CI for HR	
							Lower	Upper
Age		0.018	0.016	1.196	0.274	1.018	0.986	1.051
Gender	*Female*	0.212	0.122	3.030	0.082	1.236	0.974	1.570
Region	*Western*	0.442	0.149	8.760	0.003*	1.556	1.161	2.085
Region	*Other*	0.905	0.163	30.903	0.001*	2.471	1.796	3.400
Tertile	*1st*	0.304	0.124	6.054	0.014*	1.355	1.064	1.726
Tertile	*2nd*	0.765	0.166	21.331	0.001*	2.148	1.553	2.972
Weekdays	*Yes*	0.168	0.217	0.599	0.439	1.183	0.773	1.812
Emergency room	*Yes*	−0.126	0.147	0.736	0.391	0.881	0.660	1.176
Family Medicine	*Yes*	0.264	0.123	4.604	0.032*	1.302	1.023	1.656
Dermatology	*Yes*	− 0.128	0.200	0.406	0.524	0.880	0.594	1.304
Pediatric	*Yes*	0.012	0.136	0.008	0.931	1.012	0.775	1.321
Satellite	*Yes*	−0.203	0.275	0.545	0.460	0.816	0.476	1.399

*. Significant at α ≤ 0.05

## Results

The analysis included 264 children who had one or more scabies diagnoses at the Ministry of National Guard Health Affairs (MNGHA) hospitals and clinics across Saudi Arabia during the study period. The sample of 264 children resulted in 316 scabies diagnoses during the study period. Of 264 children, the male gender represents 54.9% (Table 1). The mean age of children was 6.7 years (±SD 3.95) with age ranges between 0.23 and 13.79 years. Of 316 scabies diagnoses, 86 (27.2%) had experienced one or more recurrent scabies with a 95% CI between 22.38 and 32.48%. Of the 86 diagnoses, the one recurrence occurred in 34 (10.8%), two recurrences occurred in 34 (10.8%), three recurrences occurred in 12 (3.8%), four recurrences occurred in 5 (1.6%), and five recurrences occurred in 1 (0.3%).

In the CPH bivariate analysis (Table 2), as compared to the Central region, the Western region of Saudi Arabia was associated with a higher risk of scabies recurrence (HR, 1.556; 95% CI, 1.161–2.085; *P* = 0.003). Compared to the 3rd tertile, 1st [January to April] (HR, 1.355; 95% CI, 1.064–1.726; *P* = 0.014) and 2nd [May to August] (HR, 2.148; 95% CI, 1.553–2.972; *P* = 0.001) tertiles were associated with an increased risk of scabies recurrence. An increased risk of scabies recurrence was observed in the Family Medicine clinic (HR, 1.302; 95% CI, 1.023–1.656; *P* = 0.032). We observed in subgroup analysis younger children (age < 6 years) reported higher prevalence of scabies recurrence (34.5%) as compared to children with 6 ≤ Age < 10 years (20.0%) and 10 ≤ Age < 14 years (21.7%), *P* = 0.024.

In the CPH multivariate analysis (Table 3), where we adjusted for confounding effects, there was a significant increase in the risk of scabies recurrence as children’s ages increased (aHR, 1.036; 95% CI, 1.002–1.072; *P* = 0.039). Females were 73.4% times more likely than males to have scabies recurrence (aHR, 1.734; 95% CI, 1.329–2.262; *P* = 0.001). The Western region of Saudi Arabia (aHR, 1.548; 95% CI, 1.115–2.151; *P* = 0.009) and 2nd tertile season [May to August] (aHR, 2.368; 95% CI, 1.706–3.288; P = 0.001) was associated with a high risk of scabies recurrence.

**Table 3:** Independent factors associated with high risk of scabies recurrence

Factor	*Levels*	B	SE	Chi-Square	P	aHR	95% CI for aHR	
							Lower	Upper
Age		0.036	0.017	4.251	0.039*	1.036	1.002	1.072
Gender	*Female*	0.550	0.136	16.464	0.001*	1.734	1.329	2.262
Region	*Western*	0.437	0.168	6.807	0.009*	1.548	1.115	2.151
Region	*Other*	0.916	0.182	25.290	0.001*	2.500	1.749	3.572
Tertile	*1st*	0.226	0.136	2.773	0.096	1.253	0.961	1.635
Tertile	*2nd*	0.862	0.167	26.527	0.001*	2.368	1.706	3.288
Weekdays	*Yes*	0.101	0.260	0.150	0.699	1.106	0.664	1.840
Emergency Room	*Yes*	0.189	0.280	0.455	0.500	1.208	0.697	2.094
Family Medicine	*Yes*	0.387	0.290	1.777	0.183	1.472	0.834	2.601
Dermatology	*Yes*	0.186	0.353	0.278	0.598	1.204	0.603	2.406
Pediatric	*Yes*	0.056	0.294	0.036	0.850	1.057	0.594	1.881
Satellite	*Yes*	0.124	0.401	0.096	0.756	1.132	0.517	2.483

*. Significant at α ≤ 0.05

## Discussion

**Fig. 1:**
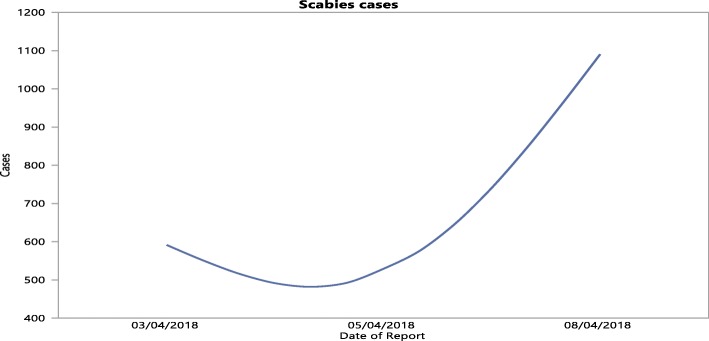
Number of cases of scabies in Mecca, Western Saudi Arabia

In the first half of 2018, outbreaks of scabies were reported in the Western region of Saudi Arabia, specifically in the Mecca area. According to Saudi health officials, the majority of the cases were identified in schools, while others were detected in homes after tracing family contacts. According to the Saudi Ministry of Health, in Mecca, the number of scabies increased to 1038 according to a report on April 5, 2018, which resulted in 419 new cases of scabies, and subsequently the number aggressively increased to 2156 on April 8, 2018, resulting in 1118 new cases of scabies (Fig. 1).

In this study, we estimated the recurrence rate of scabies in a sample of patients who received scabies diagnoses between May 20, 2015 and September 12, 2018 at the MNGHA hospitals and clinics across Saudi Arabia, as well as identifying factors for the recurrence of scabies.

As per our knowledge, in our search we identified a major gap existing in the recurrence rate of scabies among children, due to the lack of studies in most countries. The results derived from this study suggest that the recurrence of scabies was common among children in Saudi Arabia, with a rate of 27.2% who had experienced at least one or more recurrent scabies with a 95% CI between 22.38 and 32.48%. Despite the gaps in the recurrence rate of scabies among children, our estimate is in agreement with two previously reported studies among adult populations: a study in France reported 25% [7] and another study in Japan reported 32.7% [8]. This comparison has to be taken with caution as our findings were not comparable to the findings of adult studies. Recognizing the burden of this neglected tropical disease (NTD) in a prompt manner and including it on the public health agenda in Saudi Arabia and neighboring countries would increase diagnosis, proper treatment, and allow preventive measures - in addition to raising awareness and the need for education - in order to limit its spread and minimize the consequences.

The findings indicate that older children and the female gender are positively related to the recurrence of scabies. Our findings indicate that scabies recurrence tends to vary across geographical regions and the Western region of Saudi Arabia is at higher risk of scabies recurrence as compared to the country’s Central region. Associations between scabies and crowding and socioeconomic status have been reported in this region [2].

**Fig. 2:**
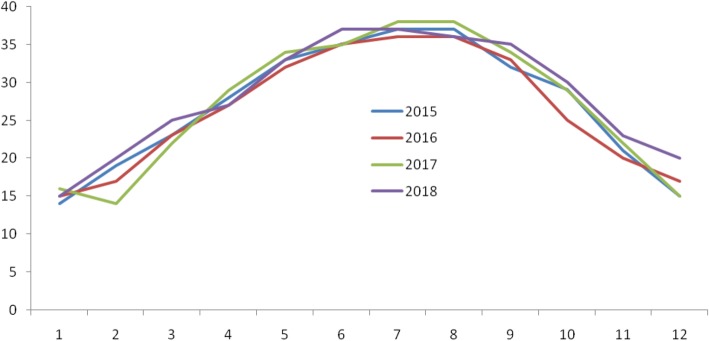
Temperature (^o^c) trends from 2015 to 2018, the highest temperature recorded in 2nd tertile- May to August

**Fig. 3:**
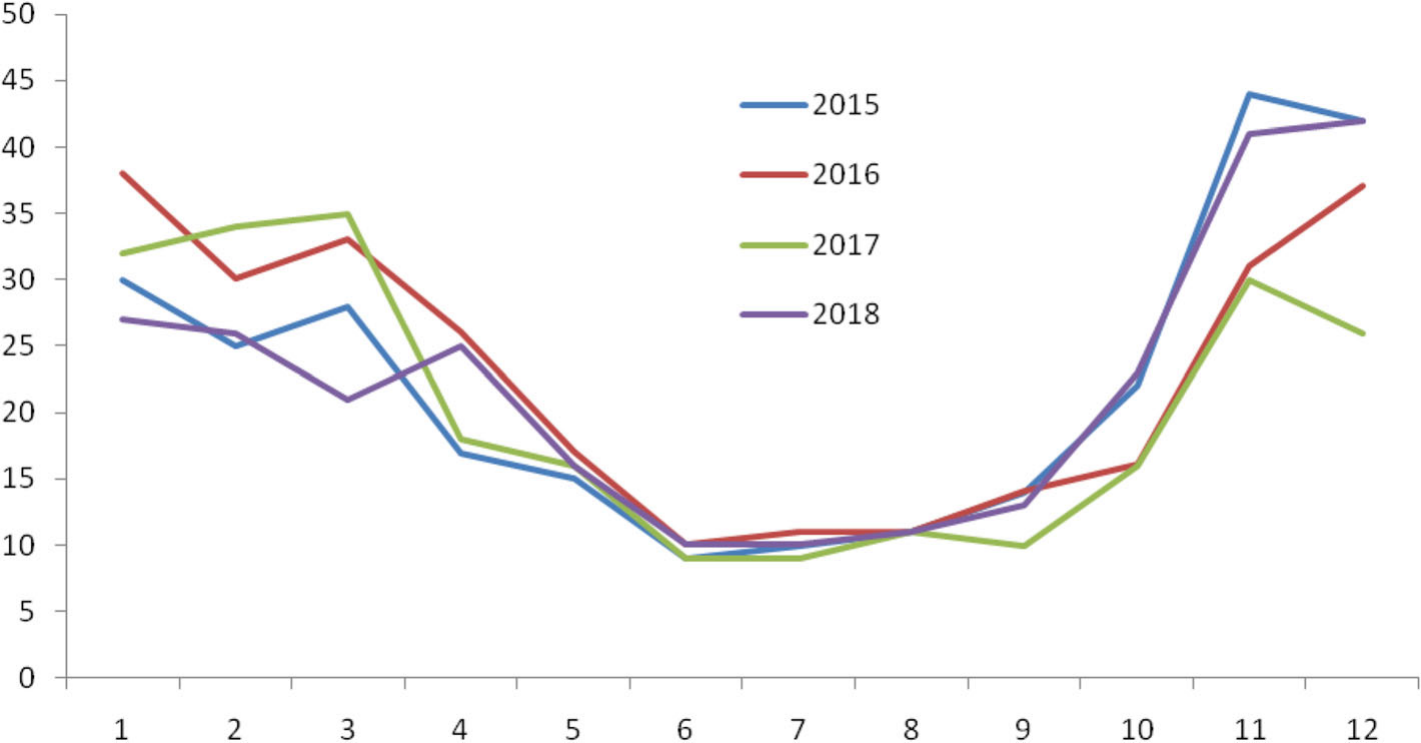
Humidity (%) trends from 2015 to 2018, the lowest humidity recorded in 2nd tertile -May to August

The recurrence rate of scabies varies according to seasons, where the highest rates were observed in the 2nd [May to August] tertile. These findings could be due to climate factors such as temperature and relative humidity as this is the period of highest temperature (^o^c) (Fig. 2) and lowest humid (%) (Fig. 3) in Saudi Arabia. Climate factors were found to have an effect on scabies infestations [15]. It would be useful to integrate the climate or seasonal factors in documenting scabies prevalence in Saudi Arabia.

The recurrence rate of scabies among children has not yet been given much attention in the literature and in our population. A number of factors may be useful to enrich surveillance MNGHA system of scabies such as condition of housing, sanitation, climate information, contacts with mites, human habitat and hygiene, and nationality or ethnic origin. The factors associated with the high risk of scabies recurrence in this study may be taken into consideration when establishing a national interventional program to prevent scabies infestations among children.

### Limitations

The retrospective design assesses associations but not causations. There is a lack of data on signs and symptoms, comorbidities, human habitat and hygiene, and proscribed treatment. Scabies diagnoses were based on MNGHA hospitals and clinics, while diagnoses occurring in another health facility were not recorded. The findings were based on a multi-center within MNGHA hospitals and clinics across Saudi Arabia. Generalization of findings may be limited to children attending MNGHA hospitals and clinics as a sample of 316 cases may not be suited to represent the Saudi general population [12].

## Conclusions

The study demonstrated that the recurrence rate of scabies among children is high. Older children, the female gender, the Western region of Saudi Arabia, and the seasonality were independently associated with an increased risk of scabies recurrence. High temperature and low humidity should be explored as leading factors for scabies infestations in Saudi Arabia. Findings derived from this study may be useful for clinicians and governments in optimizing clinical management of scabies cases and contacts.

## Data Availability

The original health records dataset pertaining to this study can be obtained from the Ministry of National Guard - Health Affairs.

## Abbreviations

aRR: Adjusted relative rate
CI: Confidence interval
MNGHA: Ministry of National Guard - Health Affairs
P: *p*-value
